# Macrophage Cx43 Is Necessary for Fibroblast Cytosolic Calcium and Lung Fibrosis After Injury

**DOI:** 10.3389/fimmu.2022.880887

**Published:** 2022-05-12

**Authors:** Aritra Bhattacharyya, Paola Torre, Preeti Yadav, Kaveh Boostanpour, Tian Y. Chen, Tatsuya Tsukui, Dean Sheppard, Rieko Muramatsu, Robert I. Seed, Stephen L. Nishimura, James B. Jung, Xin-Zi Tang, Christopher D. C. Allen, Mallar Bhattacharya

**Affiliations:** ^1^ Division of Pulmonary, Critical Care, Allergy, and Sleep, Department of Medicine, University of California, San Francisco, San Francisco, CA, United States; ^2^ Sandler Asthma Basic Research Center, University of California, San Francisco, San Francisco, CA, United States; ^3^ Cardiovascular Research Institute, University of California, San Francisco, San Francisco, CA, United States; ^4^ Department of Molecular Pharmacology, National Institute of Neuroscience, National Center of Neurology and Psychiatry, Kodaira, Japan; ^5^ Department of Pathology, University of California, San Francisco, San Francisco, CA, United States; ^6^ Department of Anatomy, University of California, San Francisco, San Francisco, CA, United States

**Keywords:** P2RX4, lung fibrosis, fibroblast, macrophage, calcium imaging

## Abstract

Macrophages are paracrine signalers that regulate tissular responses to injury through interactions with parenchymal cells. Connexin hemichannels have recently been shown to mediate efflux of ATP by macrophages, with resulting cytosolic calcium responses in adjacent cells. Here we report that lung macrophages with deletion of connexin 43 (Mac_ΔCx43_) had decreased ATP efflux into the extracellular space and induced a decreased cytosolic calcium response in co-cultured fibroblasts compared to WT macrophages. Furthermore, Mac_ΔCx43_ mice had decreased lung fibrosis after bleomycin-induced injury. Interrogating single cell data for human and mouse, we found that *P2rx4* was the most highly expressed ATP receptor and calcium channel in lung fibroblasts and that its expression was increased in the setting of fibrosis. Fibroblast-specific deletion of *P2rx4* in mice decreased lung fibrosis and collagen expression in lung fibroblasts in the bleomycin model. Taken together, these studies reveal a Cx43-dependent profibrotic effect of lung macrophages and support development of fibroblast P2rx4 as a therapeutic target for lung fibrosis.

## Introduction

Tissue fibrosis is a universal feature of the response to injury and is associated with high mortality in pulmonary fibrosis, liver cirrhosis, chronic heart failure, and chronic kidney disease. In most cases, the inflammatory response is marked by large numbers of monocyte-derived macrophages, which have been found to be necessary for lung fibrosis (1–3). Our recent work profiled the fibrotic phase of lung injury at the single-cell level, revealing a subset of *Cx3cr1+* macrophages that localize to clusters of activated fibroblasts and exert a profibrotic effect (3). With respect to the profibrotic mechanisms of these macrophages, one emerging view is that they activate surrounding fibroblasts by secreting cytokines and growth factors, such as TNFα, TGFβ, and PDGF (2–6). However, the full repertory of signals deriving from macrophages is not known.

Previous *in vitro* results have shown that extracellular ATP activates purinergic receptors on fibroblasts, leading to calcium influx and collagen expression (7, 8). ATP is a known damage associated molecular pattern that binds to and activates membrane channels for calcium entry in a range of inflammatory contexts (9). Recent reports demonstrate that connexin 43 (Cx43) hemichannels are a conduit for ATP efflux by macrophages, with paracrine effects on calcium transients in bystander cells (10–12). However, this biology has not been explored in reference to macrophage-fibroblast crosstalk.

In this study, we found lung fibroblast calcium is increased by co-culture with lung monocyte-derived macrophages. Furthermore, macrophages were found to secrete ATP in a Cx43-dependent manner after injury, and the fibroblast cytosolic calcium response was decreased in co-culture with *Cx43* KO macrophages. *In vivo* studies indicated that the ATP receptor *P2rx4* expressed by fibroblasts was necessary for lung fibrosis, revealing a novel profibrotic interaction of macrophages and fibroblasts in lung injury.

## Materials And Methods

### Mice

Ai14 (*Rosa26-LSL-tdTomato*), *Cx3cr1-CreERT2* (Cx3cr1tm2.1(cre/ERT2)Jung), *Cx43 floxed*, *Rosa26-LSL-Salsa6f*, and Macblue (Csf1r-GAL4/VP16,UAS-ECFP)1Hume/J mice were obtained from the Jackson Laboratory. *Col1a2-CreERT2* mice were obtained from Bin Zhou (13). *Pdgfrb-Cre* (14) and *P2rx4* floxed (15) mice were in the possession of the investigative team. Mice with tamoxifen-inducible alleles were administered 2 mg tamoxifen (Sigma, T5648) dissolved in olive oil (Sigma, 01514) *via* intraperitoneal injection every other day for Cre induction. All experiments were balanced for gender, and mice were used for experiments between the ages of 6 and 10 weeks. Mice were maintained in specific-pathogen-free conditions in the Animal Barrier Facility of the University of California, San Francisco.

### ATP Measurement

To isolate macrophages, we sorted tdTomato+ cells using a Sony SH 800 flow cytometer with freshly isolated lung cell suspensions from tamoxifen-induced *Cx3cr1-CreERT2: Rosa26-loxp-STOP-loxP-tdTomato* mice following lung dissociation with 2000 U/ml DNase I (Roche, 4716728001), 0.1 mg/ml Dispase II (Sigma, 4942078001) and 0.2% Collagenase (Roche, 10103586001), with gating as previously described (3). Cells were passed through a 70-µm filter prior to FACS (Sony SH800) of DAPI- (live), tdTomato+ cells followed by 24-hour culture. Supernatants were then collected for ATP measurement using a luciferase assay as described in the manufacturer’s protocol (ATP Determination Kit, ThermoFisher Scientific, A22066). Bioluminescence was quantified using a Biotek H1 Plate Reader. The ATPase inhibitor ARL 67156 (Sigma-Aldrich, 67156) was used to reduce extracellular ATP degradation.

### Macrophage-Fibroblast Co-Culture

tdTomato+ cells were isolated as above by FACS of freshly isolated cell suspensions from *Cx3cr1-CreERT2*:Ai14 or *Cx3cr1-CreERT2: Ai14: Cx43 fl/fl* mice. Primary fibroblasts were freshly isolated from mouse lung cell suspensions from *Col1a2-CreERT2: Rosa26-LSL-Salsa6f* mice by negative selection of endothelial (CD31), leukocyte (CD45), epithelial (Epcam), vascular endothelial, pericytes & smooth muscle (CD146) and red blood (Ter119) cells by biotin-labeled antibodies and Dynabeads MyOne Streptavidin T1 (Thermo Fisher Scientific, 65601) as per Tsukui et al. (16). Sorted macrophages and isolated fibroblasts were co-cultured at 1:1 in µ-slide plates (Ibidi, 81506) for 24 hours. Fluorescence images were captured by confocal microscopy with a Leica CTR 6500 microscope, and images were analyzed using Imaris software.

### Lung Slice Preparation and Calcium Imaging by Two-Photon Microscopy

Precision-cut lung slices were obtained from mouse lungs according to the protocol described (17). Mice at day 7 after injury with bleomycin were euthanized, and the trachea was cannulated followed by inflation of the lung with 1 mL of 2% low melting point agarose warmed to 37°C (Lonza, 50111) dissolved in 1X PBS. After inflation, agarose was solidified by applying ice-cold 1X PBS to the chest cavity for 1 min. Subsequently, the lungs were excised from the body along with heart and trachea and immersed in ice-cold, serum-free Leibovitz’s media (Gibco, 21083-027). Left lung lobes were isolated and precision-cut transversely in a bath of ice-cold PBS to generate 400-µm slices using a vibratome (Leica VT1200). Slices were placed in a 24-well plate in Leibovitz’s media until imaging.

During imaging, slices were housed in an HP Ultra Quiet Imaging Chamber (Warner Instruments, JG23W/HP) attached to a PM-1 Chamber platform and Series 20 stage adapter. 1X Hank’s Balanced salt solution (HBSS, Gibco, 14025-076) was bubbled with a carbogen gas mixture (95% O_2_/5% CO_2_) in a 39°C water bath and pumped with a mini-variable flow pump at a rate of approximately 1.5 mL/minute through an in-line heater into the imaging chamber, maintaining the temperature of the sample at 35-37°C with a dual-chamber temperature controller. Live calcium imaging was performed by two-photon microscopy using an upright LSM 7 MP INDIMO system (Carl Zeiss Microscopy), customized with four GaAsP detectors and a Z-Deck motorized stage (Prior). Samples were imaged with a W Plan-Apochromat 20X/1.0 N.A. water-immersion objective. To minimize the spectral overlap of CFP and GFP, these fluorophores were sequentially excited at different wavelengths. Specifically, one Chameleon Ultra II laser (Coherent) was tuned to a wavelength of 870nm to excite CFP, while the other Chameleon Ultra II laser was tuned to a wavelength of 970 nm to excite both GFP and tdTomato. Samples were imaged with two ‘tracks’ with rapid line switching of laser emissions by acousto-optic modulators (Carl Zeiss Microscopy). Emission filters were 472/30 (Semrock) for CFP, 525/50 (Chroma) for GFP, and 605/70 (Chroma) for tdTomato. Images were collected with ZEN Black software (Carl Zeiss Microscopy), and image analysis was performed with Imaris software (Bitplane). Mean GFP intensity from the entire duration of the video was plotted for all the individual tdTomato+ cells in the field of view.

### Bleomycin Lung Injury and Hydroxyproline Measurement

Animals were euthanized 21 days after injury with bleomycin (Fresenius; 3 U/kg), and lungs were perfused with 1X PBS and snap frozen in liquid nitrogen. These samples were then homogenized and incubated with 50% Trichloroacetic acid (Sigma, T6399) on ice for 20 mins followed by an overnight incubation at 110°C in 12N HCl (Fisher, A144). Samples were reconstituted in distilled water with constant shaking for 2h. Aliquots of these samples were allowed to react with 1.4% Chloramine T (Sigma, 85739) and 0.5 M sodium acetate (Sigma, 241245) in 10% 2-propanol (Fisher, A416). The samples were incubated with Ehrlich’s solution (Sigma, 03891) for 15 min at 65°C. Hydroxyproline content was measured by the analyzing absorbance at 550 nm with respect to the standard curve generated from purchased hydroxyproline (Sigma, H5534).

### Analysis of Published Mouse and Human Lung scRNAseq Data Sets

Data were downloaded from GSE132771 (16), who performed 10x-based single cell sequencing of lungs from 3 Idiopathic Pulmonary Fibrosis patients and 3 healthy controls as well as mice with and without bleomycin-induced lung injury. For human data analysis, we used the lineage-negative data from GSE132771 and combined the data with SCTransform by Seurat v3 (18). For mouse data, we used the same Seurat object of *Col1a1+* cells as Tsukui et al. (16).

### qPCR of Primary Mouse Lung Fibroblasts

Primary mouse lung fibroblasts were freshly isolated by negative selection from lung cell suspensions as above. RNA was purified using RNeasy Kit (Qiagen, 74106) with on-column DNaseI digestion. iScript reverse transcription super mix (Bio-Rad, 1708841) was used for first-strand synthesis, followed by qPCR using SYBR Green super mix (Applied Biosystems, 4309155). Gene expression was normalized to *Gapdh*. The following primers were used (5’ to 3’):

Mouse *Cx43* Forward: ACAGCGGTTGAGTCAGCTTG

Mouse *Cx43* Reverse: GAGAGATGGGGAAGGACTTGT

Mouse *Col1a1* Forward: CCTCAGGGTATTGCTGGACAAC

Mouse *Col1a1* Reverse: CAGAAGGACCTTGTTTGCCAGG

Mouse *Col3a1* Forward: GACCAAAAGGTGATGCTGGACAG

Mouse *Col3a1* Reverse: CAAGACCTCGTGCTCCAGTTAG

Mouse *P2rx4* Forward: GCTTTCAGGAGATGGCAGTGGA

Mouse *P2rx4* Reverse: TGTAGCCAGGAGACACGTTGTG

Mouse *Gapdh* Forward: AGTATGACTCCACTCACGGCAA

Mouse *Gapdh* Reverse: TCTCGCTCCTGGAAGATGGT

### ATPγS Treatment of Human and Mouse Fibroblasts

Normal adult human lung parenchyma was collected from lobectomy specimens from resections performed for primary lung cancer or from normal lungs not used for transplantation. Lung tissue was considered “normal” if the pulmonary function was normal. Informed consent was obtained from all study participants as part of an approved ongoing research protocol by the University of California San Francisco Committee on Human Research in full accordance with the declaration of Helsinki principles. Lung fibroblasts isolated were cultured by the explant technique (19) and used P1 to P4. Primary mouse lung fibroblasts were freshly isolated by negative selection from lung cell suspensions as above.

In both cases (human and mouse), cells were serum starved for 1 hour and, in some cases, treated with ATP*γ*S (Sigma, A1338). Cells were lysed, and RNA was purified using RNeasy Kit (Qiagen,74106) with on-column DNaseI digestion. iScript reverse transcription super mix (Bio-Rad, 1708841) was used for first-strand synthesis, followed by qPCR using SYBR Green super mix (Applied Biosystems, 4309155). Gene expression was normalized to *18S* rRNA.

Human *COL1A1* Forward: GATTCCCTGGACCTAAAGGTGC

Human *COL1A1* Reverse: AGCCTCTCCATCTTTGCCAGCA

Human *COL3A1* Forward: TGGTCTGCAAGGAATGCCTGGA

Human *COL3A1* Reverse: TCTTTCCCTGGGACACCATCAG

Human 18S rRNA Forward: GTAACCCGTTGAACCCCATT

Human 18S rRNA Reverse: CCATCCAATCGGTAGTAGCG

### Sirius Red With Fast Green

Staining with Sirius red (Electron Microscopy Science, 26357-02) and Fast Green FCF (Fisher Scientific, F99-10) was performed on sections prepared from mouse lungs frozen in OCT. Sections were stained with Sirius Red/Fast Green solution (0.125% Fast Green FCF and 0.1% Sirius Red in saturated Picric Acid) for 60 minutes followed by a 2-minute 0.01 N HCl wash, rinsed in 70% EtOH, and air dried overnight prior to imaging. A brightfield microscope (Zeiss Axio scan. Z1) was used for acquiring images, which were quantified using ImageJ. At least three different regions of interest were used for analysis of each sample.

### Western Blot

Fibroblasts isolated from mice were lysed with Pierce RIPA buffer (Thermo Fisher Scientific, 89901) and Halt protease inhibitor cocktail (Thermo Fisher Scientific, 1861278). 25 micrograms of protein was subjected to 10% sodium dodecyl sulfate-polyacrylamide gel electrophoresis (Bio-rad, 4561034) and transferred to a PVDF membrane (Thermo Fisher Scientific, 88520). Membranes were blocked with 5% BSA in 1X PBST (1X PBS+0.01%Tween 20) for 2h at room temperature. Thereafter, the membranes were incubated with primary antibodies: Phospho-p38 MAPK (1:1000, Cell Signaling Technology, 9211) and p38 MAPK (1:1000, Cell Signaling Technology, 9212). After washing with PBST, membranes were incubated with secondary antibody, peroxidase-conjugated goat anti-rabbit (1:20000, Anaspec, AS28177). Membranes were developed using SuperSignal West Pico Chemiluminescent substrate (Thermo Fisher Scientific, 34080) and scanned using ChemiDoc XRS+ gel imaging system (Bio-rad). Quantification of bands was done using ImageJ as described earlier (20).

### Statistical Analysis

GraphPad Prism version 8 was used for statistical analysis and linear regression. For categorical variables, Student’s t-test or Mann-Whitney test was used to calculate *P* values. For comparing more than 2 populations, 1-way ANOVA or 2-way ANOVA was used followed by Sidak’s or Kruskal Wallis multiple-comparisons testing. A *P* value of less than 0.05 was considered significant.

## Results

**Figure 1:**
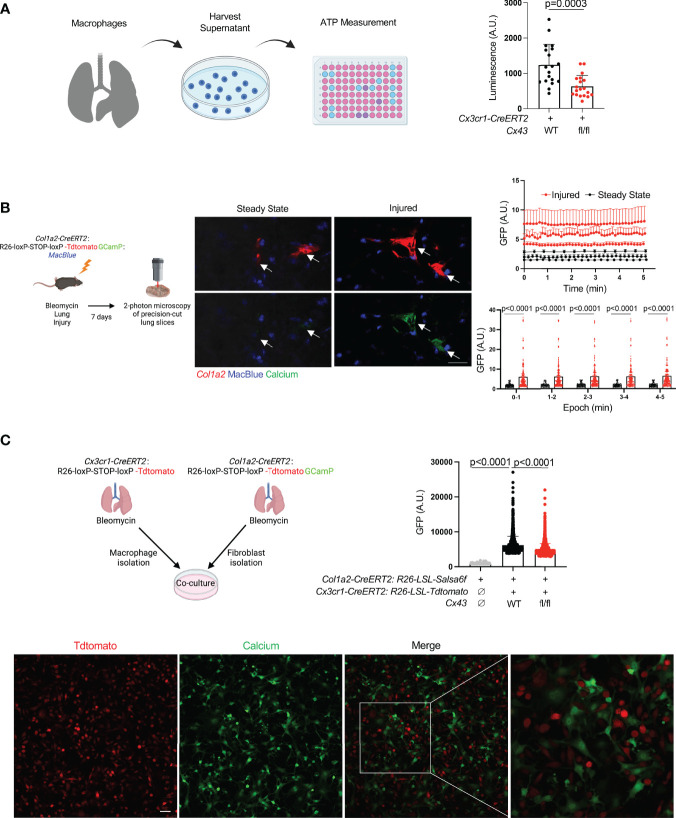
Lung moMac Cx43 is required for ATP efflux and fibroblast cytosolic calcium in co-culture. **(A)** ATP measured in conditioned media after 24 hours of culture of tdTomato+ cells sorted from tamoxifen-induced *Cx3cr1-CreERT2: LSL-tdTomat*o mice with or without homozygous floxed *Cx43* alleles. Quantitation is for n=3 mice in each group, and points in the graph represent all technical replicates. P value is for Student’s unpaired two-tailed t-test. **(B)** Representative images of two-photon calcium imaging of precision-cut lung slices taken from *Col1a2-CreERT2: R26-LSL-Salsa6f(tdtomato-GCaMP): MacBlue* mice at steady state or 7 days after bleomycin injury. Arrows indicate fibroblasts. Scale bar= 50μm. Plots are for time-lapse imaging of individual slices from n=3 mice per condition (10-18 individual cells per field of view for injured and 5-11 individual cells per field of view for steady-state slices). Upper plot shows mean cytosolic GFP fluorescence for individual fibroblasts (as marked by tdTomato) for each slice across time; lower plot presents the same data as mean GFP fluorescence for aggregate data within 5 consecutive 1-minute time periods. P values are for 2-way ANOVA followed Sidak’s multiple comparison test. **(C)** Confocal calcium imaging of macrophages sorted from tamoxifen-induced *Cx3cr1-CreERT2: R26-LSL-tdtomato* mice and co-cultured with fibroblasts isolated from tamoxifen-induced *Col1a2-CreERT2: R26-LSL-Salsa6f(tdtomato-GCaMP)* mice. A representative field of view is shown, and the right-most image magnifies the indicated area in the merge. Scale bar= 50μm. Quantitation is for co-culture with WT or Cx43 KO macrophages, or for fibroblasts alone, for n=3 mice in each condition, and points in the graph represent individual cells. P values are for one-way ANOVA followed by Sidak’s multiple comparison test.

Recent studies have shown that Cx43 hemichannels serve as a conduit for ATP release from macrophages, leading to paracrine effects on other cells in the microenvironment (10–12). To probe the potential significance of macrophage ATP efflux in fibrosis, we employed the bleomycin model of lung fibrosis (21). We first measured ATP in conditioned media of cultured tdTomato+ cells isolated from the lungs of bleomycin-injured *Cx3cr1-CreERT2: Rosa26-LSL-tdtomato* mice (largely monocytes and monocyte-derived macrophages) and found that *Cx43* KO macrophages released less ATP into the extracellular space than wild type (**Figure 1A**).

**Figure 2:**
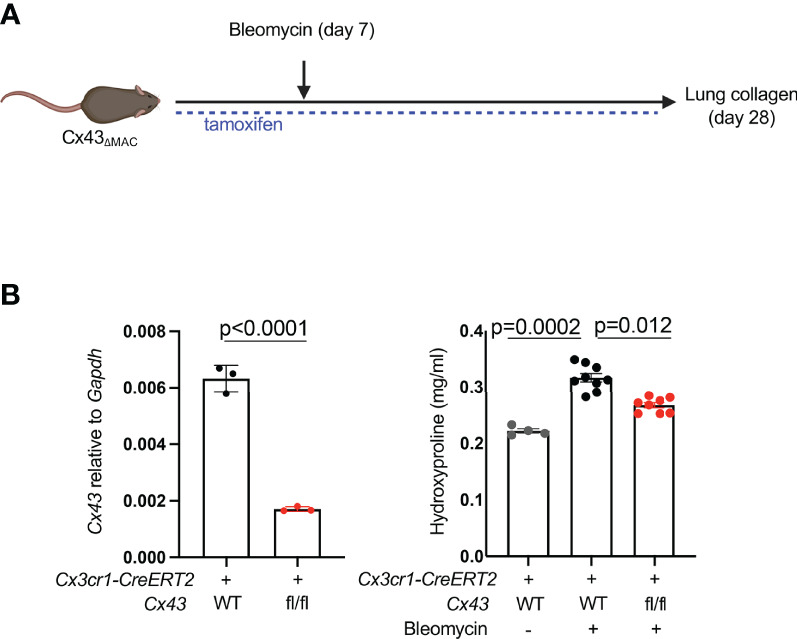
MoMac Cx43 is required for lung fibrosis. **(A)** Schematic for testing the effect of macrophage-specific Cx43 deletion on lung collagen after bleomycin injury with *Cx3cr1-CreERT2: Cx43 fl/fl* mice and controls. **(B)** qPCR of Cx43 expression in sorted tdTomato+ macrophages from *Cx3cr1-CreERT2: R26-LSL-tdtomato: Cx43 fl/fl* mice and *Cx3cr1-CreERT2: R26-LSL-tdtomato* controls. N=3 mice per condition. P value is for unpaired one-tailed t-test (left). Hydroxyproline assay of whole lung collagen from *Cx3cr1-CreERT2: Cx43 fl/fl* mice and *Cx3cr1-CreERT2* controls. N=4, 9, and 8 mice as shown. P value is for one-way ANOVA followed by Kruskal-Wallis multiple comparison test (right).

Extracellular ATP is known to bind to membrane-bound purinergic receptors that mediate extracellular calcium entry into the cytosol. First, to test whether fibroblast cytosolic calcium is elevated in injury, we imaged precision-cut lung slices by two-photon laser scanning microscopy. Slices were prepared from mice with the fibroblast-specific *Col1a2-CreERT2* (13) crossed to *R26-LSL-Salsa6f* (22), which consists of *tdTomato* fused to the calcium indicator *GCaMP*; the MacBlue allele (23) was included for visualization of moMacs. These data indicated that fibroblast cytosolic calcium was increased in injured mice compared to uninjured controls (**Figures 1B**, **Supplementary Videos 1**, **2**). We then explored the role of macrophage Cx43 by fibroblast calcium imaging in a co-culture system. Co-culture with WT macrophages was associated with higher fibroblast cytosolic calcium than co-culture with *Cx43 KO* macrophages, or fibroblasts alone (**Figure 1C**). Of note, our previous bulk RNAseq data indicated an increase in *Cx43* expression in lung monocyte-derived macrophages compared with tissue-resident alveolar macrophages after bleomycin injury (3). Therefore, to test the functional role of Cx43 in lung fibrosis resulting from injury we used the *Cx3cr1-CreERT2*, a Cre driver that has been used for functional study of monocyte-derived macrophages in multiple contexts *in vivo* (3, 24, 25). We measured lung collagen by hydroxyproline assay and found that the increase of lung collagen after bleomycin injury was markedly reduced with *Cx43* deletion in moMacs compared to wild type (**Figures 2A, B**). Taken together, these data suggest the hypothesis that macrophages efflux ATP into the extracellular space *via* Cx43, with a resulting calcium response that is necessary for lung fibrosis after injury.

**Figure 3:**
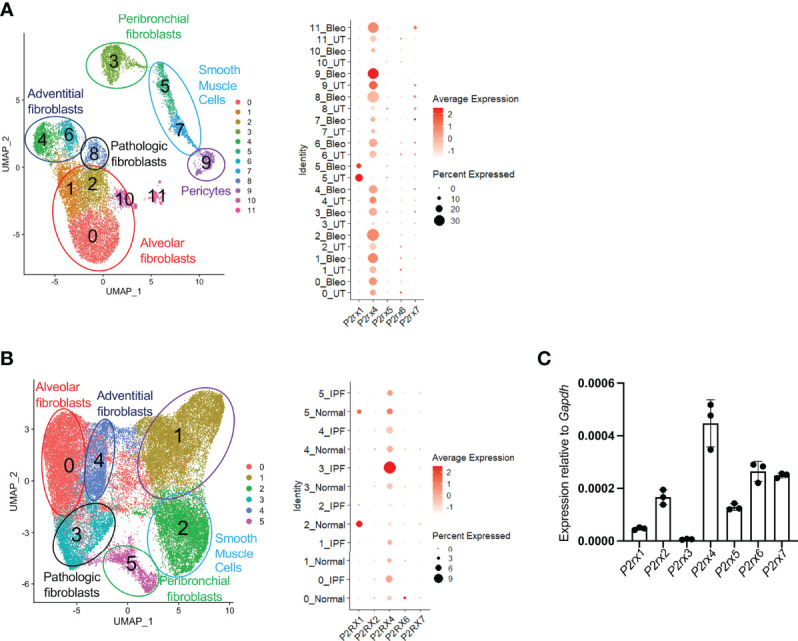
P2rx4 is the most highly expressed ionotropic ATP receptor in lung fibroblasts. **(A)** UMAP plot of mouse fibroblasts from Tsukui et al. Dot plot shows expression of ionotropic ATP receptors. **(B)** UMAP plot of human fibroblasts from Tsukui et al. Dot plot shows expression of ionotropic ATP receptors. **(C)** qPCR of ionotropic ATP receptors in primary mouse lung fibroblasts isolated at steady state. N=3 mice.

To pursue this hypothesis further, we first sought to elucidate the relevant ATP receptor in fibroblasts. We had previously identified 12 distinct subsets of collagen-producing cells from control mice and mice treated with bleomycin, as well as 6 distinct populations of collagen-producing cells from normal human lungs and lungs obtained at the time of transplant from patients with Idiopathic Pulmonary Fibrosis (16). Using these datasets, we interrogated expression of all 7 ionotropic ATP receptors in lung fibroblasts by single cell scRNAseq. We found that, among the detected ATP ionotropic receptors, *P2rx4* was the most highly expressed in nearly all populations of collagen-producing cells as measured by scRNAseq or qPCR (**Figures 3A–C**); notably, the scRNAseq data indicated that *P2rx4* was increased with bleomycin injury and in IPF lung samples relative to control. The increases in *P2rx4* were the most pronounced in the cellular subclusters previously found (16) to express the highest levels of pathologic extracellular matrix proteins (clusters 8 in mice and 3 in humans).

**Figure 4:**
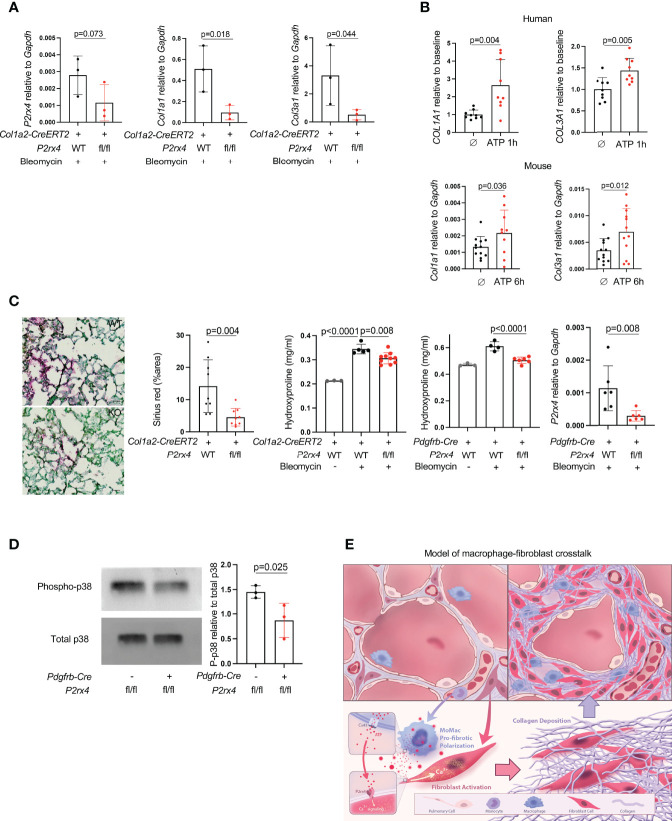
P2rx4 is necessary for lung fibrosis. **(A)** qPCR for collagen genes and *P2rx4* in primary mouse lung fibroblasts isolated from mice with fibroblast-specific *P2rx4* deletion. N=3 mice. P values are for unpaired one-tailed t-tests. **(B)** qPCR for collagen genes in human (upper) and mouse (lower) primary lung fibroblasts treated with ATPγS. N=3 separate individuals and n=3 separate mice. P values are for unpaired two-tailed t-tests. **(C)** Left: Sirius red staining of lungs at 21 days post bleomycin injury in *Col1a2-CreERT2*: *P2rx4* fl/fl or *Col1a2-CreERT2* controls. Representative images are shown. Quantification is for n=3 mice. Scale bar=50μm. P value is for unpaired two-tailed t-test. Right: Hydroxyproline assays of whole lung collagen from mice with fibroblast-specific *P2rx4* deletion with two different Cre drivers as indicated, and controls. N=3-10 as shown. P values are for one-way ANOVA followed by Sidak’s multiple comparison test. qPCR is for *P2rx4* in fibroblasts isolated from *Pdgfrb-Cre: P2rx4 fl/fl* or *Pdgfrb-Cre* controls. N=6 mice in each group and P value is for Mann-Whitney test. **(D)** Western Blot of phospho-p38 and total p38 for fibroblasts isolated from *Pdgfrb-Cre*: *P2rx4 fl/fl* or *Pdgfrb-Cre* controls. Quantification shows the ratio of P-p38 to total p38 by densitometry. P value is shown for unpaired one-tailed t-test and n=3 mice. **(E)** Model of fibroblast activation by paracrine ATP derived from monocyte-derived macrophages in lung fibrosis.

Given the high expression of *P2rx4* both at steady state and its even higher expression in pathologic fibrosis, we next tested the effect of fibroblast-specific *P2rx4* deletion by crossing a *P2rx4* floxed allele that we previously generated (15) to the *Col1a2-CreERT2* allele (13). In the entire lung fibroblast population isolated from bleomycin-injured mice, there was a modest decrease in *P2rx4* (**Figure 4A**); nonetheless, possibly because of focused expression of this collagen promoter-driven Cre in fibroblasts with high collagen production, fibroblasts from *Col1a2-CreERT2:P2rx4 fl/fl* mice had a pronounced decrease in collagen gene expression after bleomycin injury compared to wild type (**Figure 4A**). Conversely, collagen genes were induced by treatment of primary human and mouse lung fibroblasts with ATPγS (a non-hydrolyzable form of ATP; **Figure 4B**). Importantly, fibroblast-specific *P2rx4* KO mice using two different Cre drivers, the *Col1a2-CreERT2* and the *Pdgfrb-Cre*, had decreased lung collagen after bleomycin injury compared to wild type (**Figure 4C**). Moreover, we confirmed that P2rx4 signals in fibroblasts *via* p38 MAP kinase (**Figure 4D**), which has previously found to be necessary for P2rx4 signaling in other systems (26, 27). Taken together, these results suggest a model of ATP signaling *via* fibroblast P2rx4 as a positive regulator of tissue fibrosis after injury (**Figure 4E** **)**.

## Discussion

How macrophages regulate tissular remodeling in homeostasis and injury is an area of active investigation (28), and much of the focus of the recent literature has been on the ontogeny of macrophages that subserve various functions in pathologic states. A major emerging theme is the role of moMacs, which support a range of pathologic phenotypes in multiple disease models (29, 30). Lung fibrosis is no exception, with monocyte-lineage cells being shown to be associated with poor outcomes in multiple clinical cohorts (31) and in mouse models including our own prior work showing a pro-fibrotic effect of moMacs (1–3, 6). This pathologic effect of macrophages has been proposed to be related to macrophage-derived factors that both support fibroblast growth and lead to fibroblast activation, including TGFβ, PDGF, and other secreted factors (4–6).

Connexin hemichannel-mediated ATP efflux as a paracrine signaling mechanism has been described in multiple contexts (10, 11, 32). Here we found that Cx43 was necessary for lung moMac ATP efflux after bleomycin injury. Furthermore, deletion of Cx43 in moMacs decreased fibrosis, and we noted decreased cytosolic calcium in fibroblasts co-cultured with *Cx43* KO moMacs. Both human and mouse lung fibroblasts had increased expression of collagen in response to ATPγS treatment. Thus, we tested the role in lung fibrosis of ATP receptor P2rx4, which we found was the most highly expressed ionotropic ATP receptor in both murine and human lung fibroblasts. Furthermore, fibroblast *P2rx4* was upregulated in lung fibrosis and has previously been found both to have a pro-fibrotic role and to be necessary for fibroblast calcium in liver fibrosis (27). Using two different fibroblast Cre drivers, we found that fibroblast-specific deletion of *P2rx4* decreased lung collagen in mice, both at the cellular and whole-lung levels. Taken together, these results motivate enthusiasm for therapeutic development of inhibitors to P2rx4, some of which exist but are of relatively low potency (33). Moreover, they support a novel hypothesis of profibrotic macrophage-fibroblast interaction based on macrophage efflux of ATP *via* connexin hemichannels.

Our study has several limitations. While the finding of elevated fibroblast cytosolic calcium dependent on macrophage co-culture was acquired *in vitro*, importantly, we also observed injury-induced calcium elevation in fibroblasts in precision-cut lung slices, a closer reflection of the biology of intact tissue. However, future studies should seek to confirm specifically within the context of lung slice imaging the dependence of this fibroblast calcium elevation on macrophages and on extracellular ATP, since other paracrine or cell autonomous factors may be relevant. Furthermore, our *in vitro* data do not rule out the possibility that macrophage Cx43 could have other effects separate from ATP efflux, including gap junctional communication or secretion of other profibrotic mediators that may regulate cytosolic calcium in adjacent fibroblasts in a paracrine manner. We add that it remains to be determined what initiates ATP efflux by macrophages, and whether the resolution of fibrosis results from downregulation of this signaling. The use of two Cre drivers for our analysis of the effects of fibroblast P2rx4 also deserves comment. We observed modest *P2rx4* knockout in fibroblasts in aggregate with the *Col1a2-CreERT2*, which is likely due to mosaic expression with this driver, although it was reassuring to find consistent effects with the *Pdgfrb-Cre*, which achieved more marked knockout in fibroblasts in aggregate. Finally, the pathways downstream of P2rx4 that drive expression of collagen genes in fibroblasts, including the role of calcium and p38 MAP kinase signaling, will require further elucidation. Despite these limitations, our results reveal a novel role for macrophages in paracrine regulation of fibroblast calcium, as well as a specific dependency on Cx43, and raise the profile of P2rx4 as a potential therapeutic target for lung fibrosis.

## Data Availability Statement

The datasets presented in this study can be found in online repositories. The names of the repository/repositories and accession number(s) can be found in the article.

## Ethics Statement

The studies involving human participants were reviewed and approved by UCSF Institutional Review Board. The patients/participants provided their written informed consent to participate in this study. The animal study was reviewed and approved by UCSF Institutional Animal Care and Use Program.

## Author Contributions

AB, PT, and PY performed all experiments under the guidance of MB. Imaging experiments were performed by PT and AB under the direction of JJ, X-ZT, and CA. KB and TC prepared breeding and experimental stocks of genetically modified mice under the guidance of PT and MB. RM contributed *P2rx4* floxed mice. TT performed analysis of single cell RNA-seq data under the supervision of DS. RS and SN isolated and provided human lung fibroblasts for analysis. MB conceived of the work, supervised experimental planning and execution, and wrote the manuscript with input from AB, PT and RM. All authors contributed to the article and approved the submitted version.

## Funding

This work was supported by the US Department of Defense (W81XWH2110417 to MB), the National Heart, Lung, And Blood Institute (DP2HL117752 to CA), the National Institute Of Allergy And Infectious Diseases (R21AI130495 to CA), the UCSF Cardiovascular Research Institute, and the UCSF Sandler Asthma Basic Research Center.

## Conflict of Interest

The authors declare that the research was conducted in the absence of any commercial or financial relationships that could be construed as a potential conflict of interest.

## Publisher’s Note

All claims expressed in this article are solely those of the authors and do not necessarily represent those of their affiliated organizations, or those of the publisher, the editors and the reviewers. Any product that may be evaluated in this article, or claim that may be made by its manufacturer, is not guaranteed or endorsed by the publisher.

## Supplementary Material

Supplementary Video 1 5-minute time-lapse video corresponding to **Figure 1B**, with images acquired every 5 seconds by two-photon calcium imaging of precision-cut lung slices from steady-state *Col1a2-CreERT2: R26-LSL-Salsa6f(tdTomato-GCaMP): MacBlue* mice. Scale is as for **Supplementary Video 2**.

Supplementary Video 2 20-minute time-lapse video corresponding to **Figure 1B**, with images acquired every 5 seconds by two-photon calcium imaging of precision-cut lung slices from *Col1a2-CreERT2: R26-LSL-Salsa6f(tdTomato-GCaMP): MacBlue* mice 7 days post-bleomycin injury. Scale is as for **Supplementary Video 2**.
